# Endothelial Nitric Oxide Synthase Regulates White Matter Changes via the BDNF/TrkB Pathway after Stroke in Mice

**DOI:** 10.1371/journal.pone.0080358

**Published:** 2013-11-13

**Authors:** Xu Cui, Michael Chopp, Alex Zacharek, Ruizhuo Ning, Xiaoshuang Ding, Cynthia Roberts, Jieli Chen

**Affiliations:** 1 Department of Neurology, Henry Ford Hospital, Detroit, Michigan, United States of America; 2 Department of Physics, Oakland University, Rochester, Michigan, United States of America; University of South Florida, United States of America

## Abstract

Stroke induced white matter (WM) damage is associated with neurological functional deficits, but the underlying mechanisms are not well understood. In this study, we investigate whether endothelial nitric oxide synthase (eNOS) affects WM-damage post-stroke. Adult male wild-type (WT) and eNOS knockout (eNOS^-/-^) mice were subjected to middle cerebral artery occlusion. Functional evaluation, infarct volume measurement, immunostaining and primary cortical cell culture were performed. To obtain insight into the mechanisms underlying the effects of eNOS^-/-^ on WM-damage, measurement of eNOS, brain-derived neurotrophic factor (BDNF) and its receptor TrkB in vivo and in vitro were also performed. No significant differences were detected in the infarction volume, myelin density in the ipsilateral striatal WM-bundles and myelin-based protein expression in the cerebral ischemic border between WT and eNOS^-/-^ mice. However, eNOS^-/-^ mice showed significantly: 1) decreased functional outcome, concurrent with decreases of total axon density and phosphorylated high-molecular weight neurofilament density in the ipsilateral striatal WM-bundles. Correlation analysis showed that axon density is significantly positive correlated with neurological functional outcome; 2) decreased numbers of oligodendrocytes / oligodendrocyte progenitor cells in the ipsilateral striatum; 3) decreased synaptophysin, BDNF and TrkB expression in the ischemic border compared with WT mice after stroke (n = 12/group, p<0.05). Primary cortical cell culture confirmed that the decrease of neuronal neurite outgrowth in the neurons derived from eNOS^-/-^ mice is mediated by the reduction of BDNF/TrkB (n = 6/group, p<0.05). Our data show that eNOS plays a critical role in WM-damage after stroke, and eNOS^-/-^-induced decreases in the BDNF/TrkB pathway may contribute to increased WM-damage, and thereby decrease functional outcome.

## Introduction

White matter (WM) is composed of bundles of myelinated axons that connect various grey matter areas of the brain to each other [1]. Cerebral WM damage is frequently observed in human ischemic cerebrovascular disease, and is increasingly recognized as contributing to cognitive impairment and long-term disability [2], [3]. Endothelial nitric oxide synthase (eNOS) is a key target in molecular stroke research [4]. Previous studies have shown that eNOS reduces acute ischemic injury and promotes recovery following cerebral ischemia by regulation of cerebral blood flow, maintaining cerebral homeostasis, exerting anti-inflammatory effects, and by increasing angiogenesis as well as neurogenesis [5]–[8]. However, to our knowledge, there are no reports whether eNOS regulates WM changes post-stroke.

eNOS knockout (eNOS^-/-^) mice showed a reduced expression of neurotrophin brain-derived neurotrophic factor (BDNF) [5], suggesting that eNOS may impact WM by regulating BDNF. BDNF and its receptor tropomyosin-related kinase B (TrkB) have been implicated in regulating central nervous system (CNS) axon growth [9]–[11] and supporting a promyelinating role *in vivo* [12]. *In vitro* studies have shown that BDNF exerts direct effects upon oligodendroglia, variously promoting oligodendrocyte progenitor cell (OPC) proliferation and differentiation, as well as myelination via activation of endogenous TrkB receptors on oligodendroglia [13]. Thus, in this study using a model of stroke in mice, we investigate whether eNOS impacts WM-damage after stroke and the possible role of BDNF in this process.

## Materials and Methods

All experimental procedures were carried out in accordance with the NIH Guide for the Care and Use of Laboratory Animals and approved by the Institutional Animal Care and Use Committee of Henry Ford Hospital.

### Middle Cerebral Artery Occlusion (MCAo) model

Adult male C57BL/6 wild-type (WT) and eNOS^-/-^ mice (2 months old, weighting 25–30g, Jackson Laboratory) were employed in this study. All aminals were subjected to permanent right MCAo by a filament method [5]. Briefly, mice were initially anesthetized with 3.5% isoflurane and maintained with 1.0% to 2.0% isoflurane in 70% N_2_O and 30% O_2_ using a facemask. The rectal temperature was controlled at 37°C with a feedback-regulated water heating system. The right common carotid artery, external carotid artery (ECA), and internal carotid artery (ICA) were exposed. A length of 6–0 monofilament nylon suture (8.0–9.0 mm), determined by the animal weight, with its tip rounded by heating near a flame, was advanced from the ECA into the lumen of the ICA until it blocked the origin of the MCA.

### Experiment Groups

Neurological functional outcome was measured in all of the survival animals. Animals were sacrificed under deep ketamine/xylazine anesthesia at 7 days after MCAo, among which, 8 mice (n = 4 for WT-MCAo and eNOS^-/-^-MCAo, respectively) were employed for tissue protein and RNA extraction, which were used for Western blot and real-time PCR (RT-PCR) assays. The remaining 24 mice (n = 12 for WT-MCAo and n = 12 eNOS^-/-^-MCAo, respectively) were fixed by transcardial perfusion with 0.9% saline followed by 4% paraformaldehyde. The brains were then coronally sectioned, paraffin-embedded for infarct volume measurement, histochemistry and immunohistochemistry staining.

### Functional Tests

The single pellet reaching test was performed before MCAo and at 7 days after MCAo [14]. Briefly, all animals were trained 30 min daily for 5 days before MCAo, and subjected to a restricted diet overnight prior the training and experimental testing. Animals were trained to use their left forepaw to extend through the slot from Plexiglas reaching box and reach the food pellets (Bioserve Inc.). The reaching was scored as a success when the animal reached and obtained a food pellet. Otherwise, the reach was scored a miss when the animal knocked the food away or dropped the food after grasping. Each animal was provided with 20 pellets each day during the testing period. The number of the left forepaw attempts and the number of successes were counted for each animal during a 10 min testing period. Performance was defined by the success rate  =  (number of success/number of left forepaw attempts)*100. Functional evaluation was measured by an investigator who was blinded to the experimental groups.

### Histological and Immunohistochemical Assessment and Lesion Volume Measurement

The lesion volume was calculated as previously described [5]. For histological and immunohistochemical staining, a standard paraffin block was obtained from the bregma (−1 mm to +1 mm) of the brain. A series of 6 µm thick sections were cut from the block. Every 10th coronal section for a total of 5 sections was used. Histochemical-staining for Bielschowsky silver (an axon marker) and Luxol Fast Blue (LFB, a demyelination marker) [15]; immunofluorescent-staining for phosphorylated high-molecular weight neurofilament (pNFH, 1∶500; SMI31, Covance) conjugated with Cy3 (1∶200, Jackson Immunoresearch Laboratories); immunohistostaining for synaptophysin (1∶1000, Chemicon), 2′, 3′-cyclic nucleotide 3′-phosphohydrolase (CNPase, marker of mature oligodendrocytes, 1∶200, Chemicon), Platelet-derived growth factor alpha (PDGFRα, a specific marker of OPCs, 1∶400, Santa Cruz), BDNF (1∶300, Santa Cruz) and TrkB (1∶500, Santa Cruz) was performed. Control experiments consisted of staining brain coronal tissue sections as outlined above, but non-immune serum was substituted for the primary antibody.

### Immunostaining Quantification

For quantitative measurement of Bielschowsky silver, pNFH, LFB, CNPase, PDGFRα, synaptophysin, BDNF and TrkB, 5 slides from each brain with 4 fields of view on each slide from the striatum of the ischemic boundary zone (IBZ) were digitized under a 40× objective (Olympus BX40; Olympus) using a 3-CCD color video camera (Sony DXC-970MD; Sony) interfaced with an micro computer imaging device (MCID) analysis system (Imaging Research). Quantification methods included: 1) Synaptic protein, BDNF and TrkB expression - the percentage of positive area of synaptophysin, BDNF and TrkB to the total selected scan area in the IBZ; 2) Axon or myelin damage - the percentage of Bielschowsky silver-, pNFH- and LFB- positive areas to the total selected scan area in the ipsilateral striatal WM bundles in the IBZ; 3) the number of Oligodendrocytes and OPCs - the total numbers of CNPase- or PDGFRα- immunoreactive cells with the selected scan area in the 40× magnified field in the ipsilateral striatum in the IBZ were counted, and the average number of Oligodendrocytes and OPCs from 5 slides each brain with 4 fields of view on each slide were obtained.

### Primary Cortical Cell Culture

In addition to the endothelium of cerebral blood vessels, eNOS is expressed in astrocytes in the CNS [16], [17]. To investigate whether eNOS deletion decreases BDNF and TrkB expression in cultured cortical cells, we employed primary mixed cortical cell cultures containing neurons and glial cells.

Cortical cells were prepared from embryonic day 15 pregnant WT or eNOS^-/-^ mice. Briefly, embryos were removed, and the cerebral cortex dissected, stripped of meninges, and dissociated by a combination of Ca_2_/Mg_2_-free HBSS containing 0.125% trypsin digestion for 15 min. The triturated cells were passed through a 40 µm cell strainer and counted. The cells were plated in poly-D-lysine-coated (Sigma-Aldrich) dishes (35 mm, Corning) at a density of 2×10^6^ cells/ml in DMEM with 5% FBS and incubated for an initial 24 h. After 24 h, the culture medium was changed to neurobasal growth medium (Invitrogen) containing 2% B-27 (Invitrogen), 2 mM GlutaMax, and 1% antibiotic-antimycotic. Mitotic inhibitors were not added, as glial cell growth was arrested by confluence, and the paucity of growth factors in the medium used for supporting neuron survival. On day *in vitro* (DIV) 3, the culture medium was replaced with HBSS and the neurons were subjected to oxygen-glucose deprivation (OGD) for 2 h in the anaerobic chamber, and then returned to normal culture conditions. The cultures were harvested after 24 h for Western blot and RT-PCR assay.

### Neurite Outgrowth Measurement

To test whether eNOS^-/-^ decreases dendrite outgrowth and whether the mechanisms underlying the decreased neurite outgrowth are mediated by the BDNF/TrkB pathway, primary cortical cell culture was utilized. Briefly, cortical cells were plated at a density of 3×10^3^ cells/chamber in 8-chamber slides and cultured with neurobasal medium containing 2% B27 and antibiotics without mitotic inhibitors. At DIV3, the cultures were subjected to 2 h of OGD, and were grouped into (6 well/group): 1) WT-OGD; 2) eNOS^-/-^-OGD; 3) WT-OGD + K252a 200 nM (tyrosin protein kinase inhibitor, Calbiochem); 4) eNOS^-/-^-OGD + BDNF 50 ng/ml. The cultures were then returned to normal culture conditions for an additional 24 hours. For measurement of neurite outgrowth, neuron-specific class III β-tubulin (TUJ1) immunostaining was performed to present neuronal bodies and dendrites. TUJ1-fluorescently labeled neurons were photographed at 10×. Total dendrite length was measured in 20 neurons in each well using the MCID analysis system, and the total length was averaged.

### Western Blot Assay

Brain tissues extracted from the ischemic border and cortical cell cultures harvested after 24 h of OGD were used for Western blot and RT-PCR analysis. Total protein was isolated with TRIzol (Invitrogen). Specific proteins were visualized using a SuperSignal West Pico chemiluminescence kit (Pierce). Antibodies for synaptophysin (1∶1000, Chemicon), eNOS (1∶250, Cell Signaling Technology), BDNF (1∶1000; Santa Cruz), TrkB (1∶1000; Santa Cruz), myelin based protein (MBP, a myelin marker, 1∶2000, Chemicon), and β-actin (1∶2000; Sigma) were used.

### RT-PCR

The total RNA was isolated with TRIzol (Invitrogen). Quantitative PCR was performed using the SYBR Green RT-PCR method on an ABI 7000 PCR instrument (Applied Biosystems). The following primers for RT-PCR were designed using Primer Express software (ABI). BDNF Fwd: TAC TTC GGT TGC ATG AAG GCG; Rev: GTC AGA CCT CTC GAA CCT GCC. TrkB Fwd: TCA TCA AGT CAG AGG TGA CAG G; Rev: ACT GGG TAC ACT CCT TCT CTC G. GAPDH: Fwd: AGA ACA TCA TCC CTG CAT CC; Rev: CAC ATT GGG GGT AGG AAC AC. Each sample was tested in triplicate, and samples were obtained from six independent experiments that were used for analysis of relative gene expression data using the 2^−ΔΔCT^ method.

### Statistical Analysis

Independent two-sample t-test was used to assess the neurological functional outcome, lesion volume, immunostaining, Western blot and RT-PCR measurement. Correlations between the success of single pellet reaching and the density of Bielshowsky silver-stained axons were tested by Pearson's correlation coefficients. One-way ANOVA and Tukey test after Post Hoc test were performed for analyzing neurite outgrowth from cortical neurons. All data are presented as mean ± Standard Error (SE). Statistical analysis was performed in a blinded manner.

## Results

### Mortality Rate

Within 7 days after stroke, 9 mice died out of the 41 subjected to MCAo (3 in 19 WT group and 6 in 22 eNOS^-/-^ group). The mortality rate in eNOS^-/-^ mice with stroke (27.3%) was significantly higher than in WT stroke mice (15.8%).

### Lesion Volume and Neurological Functional Outcome

**Figure 1:**
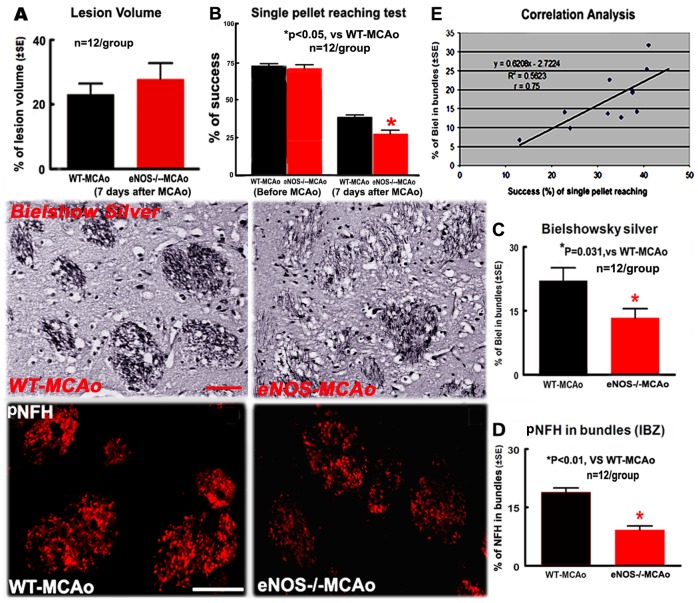
eNOS^-/-^ increased neurological functional deficits and axon damage, but not increased lesion volume in the ischemic brain 7 days after stroke. Axon density is positively correlated with the functional outcome. A: Lesion volume; B: Single pellet reaching test; C: Bielshowsky silver- staining in the bundles of striatum and quantitative data; D: pNFH-immunostaining in the striatal bundles and quantitative data. E: Correlation analysis between single pellet reaching test and axon density. Scale bar in C = 50 µm; in D = 25 µm. *p<0.05, n = 12/group

No significant difference was found in the ischemic lesion volume between WT-MCAo and eNOS^-/-^-MCAo groups (Fig.1A, p = 0.436, n = 12/group).

The single pellet reaching test measures the ability of skilled forepaw use [18]. There was no significant difference in the percentage of successful single pellet reaching between WT and eNOS^-/-^ mice prior to MCAo, but the degree of functional deficits in eNOS^-/-^ mice tested 7 days after MCAo was significantly worse than WT mice (Fig.1B, p<0.05, n = 12/group). Independent two-sample t-test was used for the statistical analysis of lesion volume and functional outcome.

### eNOS^-/-^ Increased WM-damage after MCAo

Compared with the WT-MCAo mice, eNOS^-/-^-MCAo mice exhibited a significant decrease in the density of Bielschowsky sliver-stained axons (Fig.1C, p = 0.031) and the density of pNFH-immunoreactive neurofilament (Fig.1D, p<0.01) in the ipsilateral striatal bundles in the IBZ (n = 12/group). Correlation analysis showed that the success rate of single pellet reaching was significantly positive correlated with the density of Bielschowsky sliver-stained axons (Fig.1E, r = 0.75).

**Figure 2:**
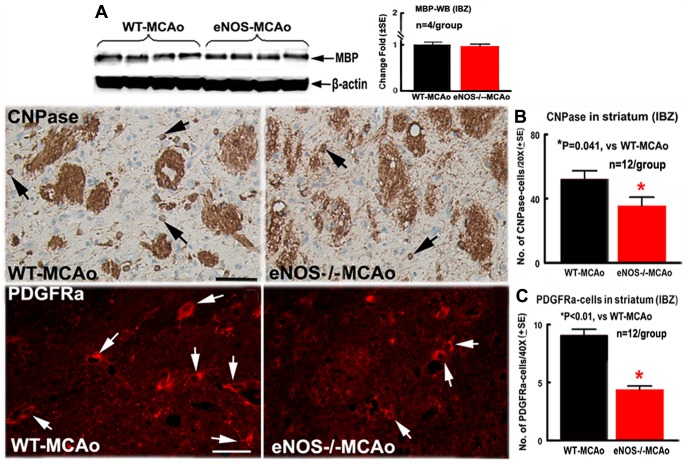
eNOS^-/-^ decreased the number of oligodendrocytes and OPCs but not demyelination in the ischemic striatal bundles 7 days after stroke. A: MBP protein expression measured by Western blot and quantitative data; B: CNPase-immunostaining and quantitative data; C: PDGFRα-immunostaining and quantitative data. Scale bar in B = 50 µm, in C = 25 µm. *p<0.05, n = 4/group in A; n = 12/group in B and C.

There was no significant difference in the density of LFB-stained myelin in the ipsilateral striatal bundles between WT-MCAo and eNOS^-/-^-MCAo mice (WT-MCAo: 25.21%±3.64%; eNOS^-/-^-MCAo: 21.39%±6.29%, p = 0.260). Western blot analysis also showed that the MBP protein level in the ischemic brain did not decrease at 7 days after MCAo (Fig.2A) in eNOS^-/-^-MCAo mice compared to WT-MCAo mice. However, eNOS^-/-^-MCAo mice exhibited a significantly decreased number of CNPase-immunoreactive oligodendrocytes (Fig.2B, p = 0.041) and PDGFRα-immunoreactive OPCs (Fig.2C, p<0.01) in the ischemic striatal border compared with WT-MCAo mice (n = 12/group). Taken together, these data indicate that compared with WT-MCAo mice, the eNOS^-/-^-MCAo mice did not exhibit increased demyelination (LFB-myelin and MBP) but demonstrated significantly increased axon damage and decreased the numbers of oligodendrocytes and OPCs.

Independent two-sample t-test was used for the statistical analysis of WM-damage between WT-MCAo and eNOS^-/-^-MCAo mice. Correlations between the success of single pellet reaching and the density of Bielshowsky silver-stained axons were tested by Pearson's correlation coefficients.

### eNOS^-/-^ Decreases Synaptic Protein Expression after MCAo

**Figure 3:**
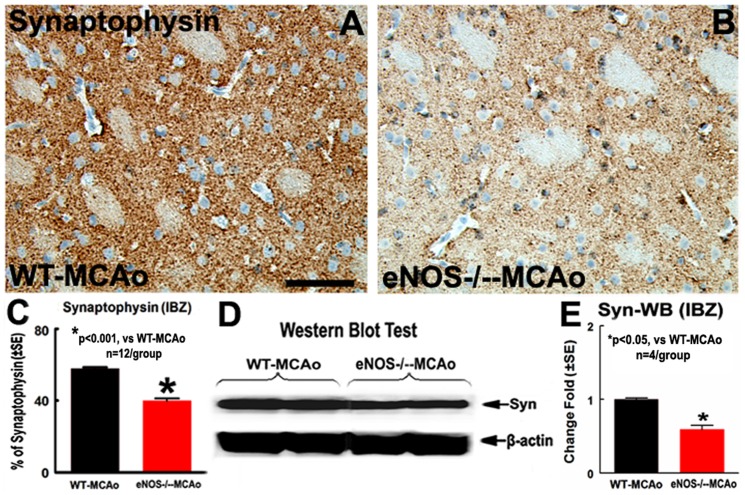
eNOS^-/-^ decreased synaptic protein expression in the ischemic brain. A–C: Synaptophysin-immunostaining and quantitative data; D and E: Synaptophysin-Western blot and quantitative data. Scale bar in A = 50 µm, *p<0.001, n = 12/group in A–C; *p<0.05, n = 4 in D and E.

To further elucidate whether eNOS^-/-^ decreases synaptic protein after stroke, synaptophysin protein expression was measured by immunostaining and Western blot analysis. eNOS^-/-^-MCAo mice exhibit significantly decreased synaptophysin expression in the ipsilateral ischemic border compared with WT-MCAo mice (Fig.3A–C, p<0.001, n = 12/group). Western blot assay also showed eNOS^-/-^ significantly decreased synaptophysin protein levels in the IBZ (Fig.3D, p<0.05, n = 4/group). These data suggest that eNOS^-/-^ mice have decreased synaptic protein expression in the ischemic brain after stroke compared with WT mice. Independent two-sample t-test was used for the statistical analysis of synaptophysin between WT-MCAo and eNOS^-/-^-MCAo mice.

### eNOS^-/-^ Mice Exhibit Decreased BDNF/TrkB Expression in the Ischemic Brain

**Figure 4:**
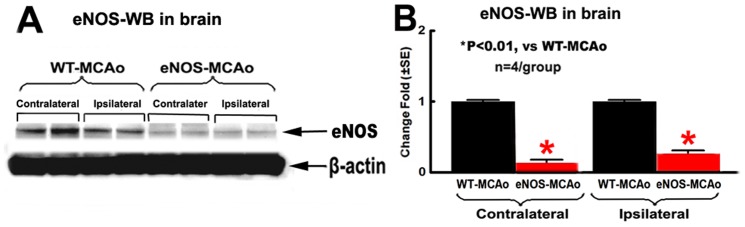
eNOS^-/-^ mice show significantly decreased eNOS expression in both ischemic ipsilateral and contralateral brain. A: eNOS Western blot; B: quantitative data of eNOS-Western blot assay. *p<0.01, n = 4/group.

**Figure 5:**
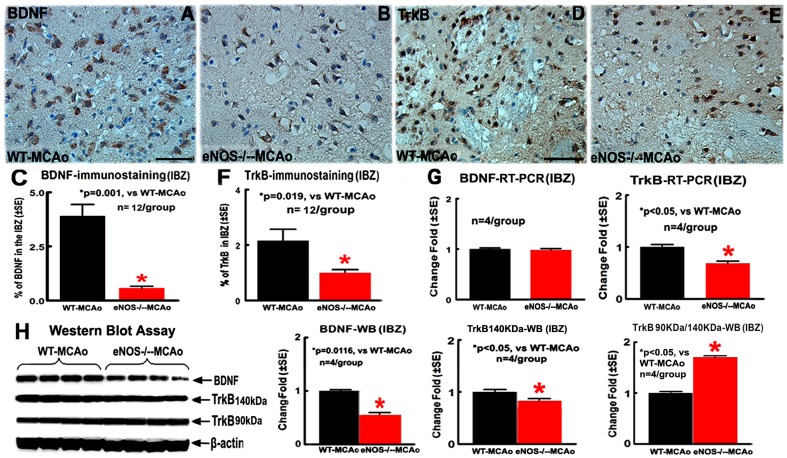
eNOS^-/-^ mice show significantly decreased BDNF and TrkB expression in the ischemic brain. A-C: BDNF-immunostaining and quantitative data in the IBZ; D-F: TrkB-immunostaining and quantitative data in the IBZ; G: Quantitative data of BDNF/TrkB-mRNA expression in the IBZ. H: BDNF, 140 KDa and 90 KDa of TrkB protein expression in the IBZ measured by Western blot and quantitative data; Scale bar in A = 50 µm; *p<0.05, n = 12/group in A–F, n = 4/group in G and H.

To investigate the mechanism of eNOS^-/-^ increased WM damage, BDNF/TrkB and eNOS expression in the brain were measured. Decreased eNOS-expression in both ischemic ipsilateral and contralateral brain of eNOS^-/-^ mice was confirmed by Western blot assay (Fig.4, p<0.05, n = 4/group). eNOS^-/-^-MCAo mice exhibit significantly decreased BDNF protein expression in the ipsilateral IBZ, measured by immunostaining and Western blot analysis (Fig.5A–C and H, p<0.05, n = 12/group for immunostaining; n = 4/group for Western blot), but BDNF mRNA level measured by RT-PCR was negative (Fig.5G, n = 4/group), compared with WT-MCAo mice.

TrkB protein expression measured by immunostaining and TrkB mRNA expression in the IBZ were also significantly decreased in eNOS^-/-^-MCAo mice compared with WT-MCAo mice (Fig.5D–G, p<0.05, n = 12/group for immunostaining; n = 4/group for Western blot). There are two major isoforms of TrkB, full-length functional (140 KDa) and truncated non-functional (90 KDa) isoforms, and they exhibit different characteristics. The production of truncated isoform of TrkB receptors is induced in response to an injury [19]. Therefore, increased truncated TrkB receptors also contribute to BDNF-TrkB signaling in neuronal injury. We examined the expression of both full-length and truncated TrkB by Western blot. As shown in Fig.5H, the expression of 140 kDa TrkB in the IBZ in eNOS^-/-^-MCAo mice was significantly decreased; however, the relative levels of 90 kDa TrkB compared to 140 kDa TrkB (Ratio of 90/140 kDa TrkB) was significantly increased compared with WT-MCAo mice (p<0.05, n = 4/group). These data indicate that eNOS^-/-^-MCAo mice exhibit decreased full-length BDNF, but increased truncated BDNF compared with WT mice 7 days after stroke.

Independent two-sample t-test was used for the statistical analysis of BDNF and TrkB expression between WT-MCAo and eNOS^-/-^-MCAo mice.

### eNOS^-/-^ Decreased BDNF/TrkB Expression and Neurite Outgrowth in Cortical Cell Cultures Derived from eNOS^-/-^ Mice after OGD

**Figure 6:**
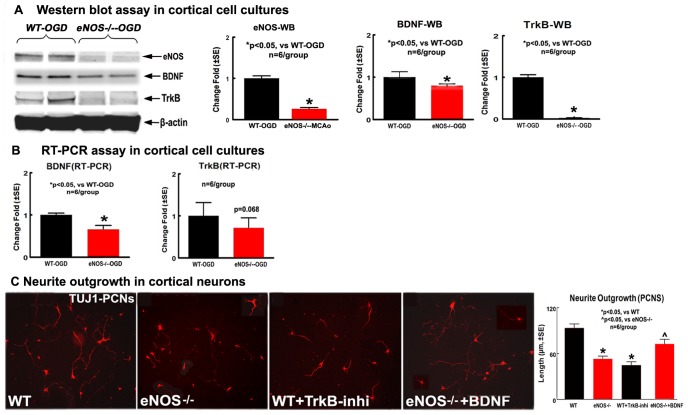
BDNF/TrkB mediates eNOS^-/-^ -induced neurite outgrowth reduction after OGD in cortical cell cultures. A: Western blot data for eNOS, BDNF and TrkB protein level; B: RT-PCR data for BDNF and TrkB mRNA level. C: Neurite outgrowth and quantitative data in cultured cortical neurons. *p<0.05, n = 6 well/group.

To complement the in vivo data that eNOS^-/-^ mice exhibit decreased BDNF/TrkB expression and increased WM-damage, the BDNF and TrkB protein and mRNA expression in the cortical cell cultures (containing neurons, asctrocytes and oligodendrocytes) were measured by Western blot and RT-PCR assays. Fig.6A shows that eNOS, BDNF and TrkB protein expression were significantly decreased in the eNOS^-/-^-cortical cell cultures compared with in the WT-cortical cell cultures (p<0.05, n = 6/group). Fig.6B shows BNDF mRNA level was significantly decreased (p<0.05, n = 6/group) and TrkB mRNA level was marginally (p = 0.068, n = 6/group) decreased in the eNOS^-/-^-cortical cell cultures compared with WT-cortical cell cultures. Independent two-sample t-test was used for the statistical analysis of BDNF and TrkB expression between the cortical cell cultures derived from WT and eNOS^-/-^ mice, respectively.

To further test whether BDNF/TrkB mediates the eNOS^-/-^ induced reduction of neurite outgrowth, neurite outgrowth was measured in cortical neurons. Fig.6C shows the neurite outgrowth significantly decreased in eNOS^-/-^-neurons compared to WT-neurons, and inhibition of TrkB in WT-neurons significantly decreased neurite outgrowth compared with the non-treatment WT-neurons (p<0.05, n = 6/group). In addition, BDNF treatment in eNOS^-/-^-neurons significantly attenuated the eNOS^-/-^ induced decreased neurite outgrowth compared with the eNOS^-/-^-neurons. eNOS is not only expressed in cerebral vascular endothelial cells, but also in astrocytes [16]. One-way ANOVA and Tukey test after Post Hoc test were performed for analyzing neurite outgrowth from cortical neurons.

Taken together, these data indicate that eNOS^-/-^ significantly decreased BDNF expression in astrocytes, and the low BNDF secretion from astrocytes may further decrease TrkB level in neurons and thereby decrease neurite outgrowth. Therefore, eNOS^-/-^-induced reduction of neurite outgrowth is, at least partially, mediated by BDNF/TrkB pathway.

## Discussion

eNOS plays a crucial role in vascular function and neuroprotection from ischemic stroke [20], [21]. eNOS is predominantly expressed by vessels endothelial cells [22]–[24] and are also located in Purkinje cell bodies in the cerebellar cortex, olfactory bulb, dentate nucleus in granular layer and hippocampal pyramidal cells, and astrocytes surrounding the cerebral blood vessels [16], [17], [25]–[27]. Astrocytes detect neuronal activity and can release neurotransmitters, which in turn control synaptic activity [28]–[31]. eNOS-positive astrocytic perisynaptic sheaths on neuronal somas in the cortex may influence neuronal transmission directly at axon-somatic synapses in the cortex [16]. In addition, the presence of eNOS in astrocytes and in their processes that contact blood vessels suggests that the link between local cortical activity and changes in cerebral blood flow could be mediated by astrocytic release of nitric oxide [16].

There is substantial evidence that many of the therapeutic approaches to the treatment of stroke, such as statins, rosiglitazone and physical activity, exert their effects by increasing eNOS [4], [21], [32], [33]. Simvastatin treatment of stroke protects against cerebral injury by upregulating eNOS, increasing functional protein expression, and augmentation of cerebral blood flow. However, the effects of simvastatin are completely absent in eNOS^-/-^ mice [21], [34]. Mevastatin, also increased levels of eNOS mRNA and protein, reduced infarct size, and improved neurological deficits in a dose- and time-dependent manner in mice [32]. eNOS also mediates T090317, a liver X receptor agonist, treatment-induced angiogenesis and improved functional outcome after stroke in mice [6]. Voluntary physical activity improves long-term stroke outcome by eNOS-dependent mechanisms related to improved angiogenesis and cerebral blood flow [33]. These findings support the importance of targeting eNOS as a means to induce stroke protection and enhance postischemic neuroregeneration [17]. In this study, we extend the role of eNOS in mediating neuroprotection. We demonstrate that eNOS^-/-^ mice exhibit no significant difference in lesion volume 7 days after MCAo but increased mortality compared with WT mice, these data are consistent with our previously reported studies [5], [35]. However, eNOS^-/-^ mice exhibit more severe WM alterations, including decreases in phosphorylated-neurofilament and axon density, and neurological functional outcome after focal brain ischemia compared to WT mice. Moreover, axonal damage is positively significantly correlated with the functional outcome. Synaptophysin is an indicator of presynaptic plasticity and synaptogenesis [36]. Dysregulation of synaptogenesis also plays an important role in the development of pathologies associated with stroke [3], [37]. We also observed that synaptophysin, a synaptic protein, is significantly decreased in eNOS^-/-^ mice.

Oligodendrocytes are responsible for the formation of myelin sheaths surrounding axons, and myelin acts as an insulator, increasing the speed of transmission of all nerve signals [38]. Following cerebral ischemia, WM damage identified by a decrease in synapses, axonal density and myelination, is coincident with decreases of neural progenitor cells and OPCs [37], [39]–[41]. In the present study, there was no significant difference in demyelination presented by LFB-stained myelin density and MBP expression between WT-MCAo and eNOS^-/-^-MCAo mice; however, eNOS^-/-^ mice exhibited reduced numbers of oligodendrocytes/OPCs post stroke. This might result because oligodendrocytes and OPCs are particularly sensitive to ischemic insult [42]. Stroke induces significantly oligodendrocyte and OPC death 7 days after stroke [43]. However, demyelination usually does not change much in the early stage after ischemia, but is significantly decreased 10 days to 56 days after stroke [44]. Our data indicate, for the first time, eNOS not only regulates vascular changes and neurogenesis, but eNOS plays a vital role in mediating WM integrity and function alterations after stroke.

BDNF is the most abundant neurotrophin in the brain and mediates axon growth and brain plasticity [9]. BDNF knockout mice show significant synaptic fatigue, and exogenous BDNF attenuates synaptic fatigue [45], [46]. Stroke induces an increase in mature BDNF expression which coincides with the increase of synaptophysin both in ipsilateral cortex and hippocampal territories [47]. Post-stroke treatment with amphetamine facilitates behavioral recovery, which is associated with an increase in synaptogenesis and upregulation of BDNF in the lesioned cortex [48]. Voluntary exercise not only upregulates eNOS, but also leads to an endogenous upregulation of BDNF and associated proteins involved in synaptic function, and enhances functional recovery after traumatic brain injury [49]. Targeted deletion of TrkB by the nestin promoter show significant reduction in cortical myelin expression (MBP and CNPase), the number of myelinated axons, and the thickness of myelin sheath in the corpus callosum [50]. We have previously demonstrated that eNOS^-/-^ mice exhibit reduced expression of brain BDNF, eNOS by regulating BDNF expression is also a critical mediator of neurogenesis in the brain, and the reduction of BDNF in eNOS^-/-^ mice may be responsible for the deficits in functional recovery and reduced brain plasticity [5]. In the present study, we found that eNOS^-/-^ mice showed a significant decrease in BDNF, 140KDa TrkB and relative level of 90KDa/140KDa TrkB, concurrently with a decrease in axon density and synaptophysin, and functional outcome. Our new data in concert the other reports, indicate that eNOS plays an important role in regulating endogenous BDNF and TrkB, which are involved in axon growth and synaptic function, and thereby in functional recovery after stroke.

Under physiological conditions, BDNF is predominantly synthesized and secreted from pre- and postsynaptic neurons, whereas astrocytes have a role in its storage and secretion [51]–[53]. Growing evidence suggests that astrocytes synthesize BDNF after induction of a neural lesion [54] or inflammation [51]. The cerebral microvasculature has also been identified as a source of BDNF that can influence the generation and survival of neurons, and brain-derived endothelial cells under hypoxia exhibit increased secretion of BDNF [55], [56]. BDNF is secreted into extracellular and then binds to its cell surface recepters TrkB or P^75^ to activate its function [9], [57]. In addition, BDNF exerts its effects on oligodendroglia via activation of TrkB receptors [13], [58], [59]). Using co-cultures of dorsal root ganglia neurons and oligodendrocyte precursor cells, phosphorylation of TrkB was highly correlated with myelination, and inhibiting TrkB signalling also inhibited the promyelinating effect of BDNF, suggesting that BDNF enhances CNS myelination via activating oligodendrocyte TrkB-full length receptors [13]. *In vitro* analyses of basal forebrain-derived OPCs reveal that BDNF increases their proliferation as well as their differentiation into mature oligodendrocytes [58]–[60]. In the present study, BDNF/TrkB expression and neurite outgrowth reduction were also found in primary cortical cell cultures derived from eNOS^-/-^ mice. Blocking TrkB decreased neurite outgrowth in WT-cortical neurons, and exogenous administration of BDNF attenuates neurite outgrowth reduction in eNOS^-/-^–cortical neurons. Our in vitro data also support the hypothesis that the increased WM damage post-stroke observed in eNOS^-/-^ mice is at least partially, mediated by decreasing BDNF/TrkB signaling activity. From our data, BDNF-mRNA in the ischemic brains and TrkB-mRNA in the cortical cell cultures derived from eNOS^-/-^-MCAo mice were not significantly decreased compared to WT-MCAo mice. RT-PCR measures transcription, and mRNA presence is not sufficient to confirm the expression of the corresponding mature/active protein since protein expression is also regulated by post-translational regulation.

In summary, our data indicate that eNOS^-/-^ mice exhibit increased neurological functional deficits after stroke. The concomitantly increased WM damage and decreased synaptic protein as well as reduction in the numbers of oligodendrocytes/OPCs may contribute to worsening of neurological functional deficits observed in eNOS^-/-^ mice. Thus, the BDNF/TrkB pathway, at least partially, mediates eNOS^-/-^ reduced WM damage after stroke.

## References

[1] Persson LA (1981) Growth of nerve-cell body and myelinogenesis in mouse trigemnal ganglion and root: a combined cytofluorometric and morphometric study. J Neurocytol 10: 169–182. 7310449 10.1007/BF01257965

[2] Assaf Y, Pasternak O (2008) Diffusion tensor imaging (DTI)-based white matter mapping in brain research: a review. J Mol Neurosci 34: 51–61. 18157658 10.1007/s12031-007-0029-0

[3] Sozmen EG, Kolekar A, Havton LA, Carmichael ST (2009) A white matter stroke model in the mouse: axonal damage, progenitor responses and MRI correlates. J Neurosci Methods 180: 261–272. 19439360 10.1016/j.jneumeth.2009.03.017 PMC4697458

[4] Endres M, Laufs U, Liao JK, Moskowitz MA (2004) Targeting eNOS for stroke protection. Trends Neurosci 27: 283–289. 15111011 10.1016/j.tins.2004.03.009

[5] Chen J, Zacharek A, Zhang C, Jiang H, Li Y, et al (2005) Endothelial nitric oxide synthase regulates brain-derived neurotrophic factor expression and neurogenesis after stroke in mice. J Neurosci 25: 2366–2375. 15745963 10.1523/JNEUROSCI.5071-04.2005 PMC2791344

[6] Chen J, Cui X, Zacharek A, Roberts C, Chopp M (2009) eNOS mediates TO90317 treatment-induced angiogenesis and functional outcome after stroke in mice. Stroke 40: 2532–2538. 19443804 10.1161/STROKEAHA.108.545095 PMC2724074

[7] Chen MJ, Ivy AS, Russo-Neustadt AA (2006) Nitric oxide synthesis is required for exercise-induced increases in hippocampal BDNF and phosphatidylinositol 3′ kinase expression. Brain Res Bull 68: 257–268. 16377431 10.1016/j.brainresbull.2005.08.013

[8] Murohara T, Asahara T, Silver M, Bauters C, Masuda H, et al (1998) Nitric oxide synthase modulates angiogenesis in response to tissue ischemia. J Clin Invest 101: 2567–2578. 9616228 10.1172/JCI1560 PMC508846

[9] Huang EJ, Reichardt LF (2001) Neurotrophins: roles in neuronal development and function. Annu Rev Neurosci 24: 677–736. 11520916 10.1146/annurev.neuro.24.1.677 PMC2758233

[10] Cui X, Chopp M, Shehadaha A, Zachareka A, Nicholsc NK, et al.. (2012) Therapeutic benefit of treatment of stroke with Simvastatin and human umbilical cord blood cells: neurogenesis, synaptic plasticity and axon growth. Cell Transplant. 10.3727/096368911X627417 PMC3442771 22405262

[11] Runyan SA, Phelps PE (2009) Mouse olfactory ensheathing glia enhance axon outgrowth on a myelin substrate in vitro. Exp Neurol 216: 95–104. 19100263 10.1016/j.expneurol.2008.11.015 PMC2821080

[12] Djalali S, Holtje M, Grosse G, Rothe T, Stroh T, et al (2005) Effects of brain-derived neurotrophic factor (BDNF) on glial cells and serotonergic neurones during development. J Neurochem 92: 616–627. 15659231 10.1111/j.1471-4159.2004.02911.x

[13] Xiao J, Wong AW, Willingham MM, van den Buuse M, Kilpatrick TJ, et al (2010) Brain-derived neurotrophic factor promotes central nervous system myelination via a direct effect upon oligodendrocytes. Neurosignals 18: 186–202. 21242670 10.1159/000323170

[14] Liu Z, Li Y, Zhang RL, Cui Y, Chopp M (2011) Bone marrow stromal cells promote skilled motor recovery and enhance contralesional axonal connections after ischemic stroke in adult mice. Stroke 42: 740–744. 21307396 10.1161/STROKEAHA.110.607226 PMC3060040

[15] Linares D, Taconis M, Mana P, Correcha M, Fordham S, et al (2006) Neuronal nitric oxide synthase plays a key role in CNS demyelination. J Neurosci 26: 12672–12681. 17151270 10.1523/JNEUROSCI.0294-06.2006 PMC6674851

[16] Wiencken AE, Casagrande VA (1999) Endothelial nitric oxide synthetase (eNOS) in astrocytes: another source of nitric oxide in neocortex. Glia 26: 280–290. 10383047

[17] Dinerman JL, Dawson TM, Schell MJ, Snowman A, Snyder SH (1994) Endothelial nitric oxide synthase localized to hippocampal pyramidal cells: implications for synaptic plasticity. Proc Natl Acad Sci U S A 91: 4214–4218. 7514300 10.1073/pnas.91.10.4214 PMC43755

[18] Farr TD, Whishaw IQ (2002) Quantitative and qualitative impairments in skilled reaching in the mouse (Mus musculus) after a focal motor cortex stroke. Stroke 33: 1869–1875. 12105368 10.1161/01.str.0000020714.48349.4e

[19] Frisen J, Verge VM, Fried K, Risling M, Persson H, et al (1993) Characterization of glial trkB receptors: differential response to injury in the central and peripheral nervous systems. Proc Natl Acad Sci U S A 90: 4971–4975. 8389459 10.1073/pnas.90.11.4971 PMC46635

[20] Matthias GKaE (2008) eNOS And Stroke: Prevention, Treatment And Recovery 537–550 p.

[21] Laufs U, Endres M, Stagliano N, Amin-Hanjani S, Chui DS, et al (2000) Neuroprotection mediated by changes in the endothelial actin cytoskeleton. J Clin Invest 106: 15–24. 10880044 10.1172/JCI9639 PMC314365

[22] Bouloumie A, Schini-Kerth VB, Busse R (1999) Vascular endothelial growth factor up-regulates nitric oxide synthase expression in endothelial cells. Cardiovasc Res 41: 773–780. 10435050 10.1016/s0008-6363(98)00228-4

[23] Yuhanna IS, MacRitchie AN, Lantin-Hermoso RL, Wells LB, Shaul PW (1999) Nitric oxide (NO) upregulates NO synthase expression in fetal intrapulmonary artery endothelial cells. Am J Respir Cell Mol Biol 21: 629–636. 10536122 10.1165/ajrcmb.21.5.3749

[24] Zheng J, Bird IM, Melsaether AN, Magness RR (1999) Activation of the mitogen-activated protein kinase cascade is necessary but not sufficient for basic fibroblast growth factor- and epidermal growth factor-stimulated expression of endothelial nitric oxide synthase in ovine fetoplacental artery endothelial cells. Endocrinology 140: 1399–1407. 10067868 10.1210/endo.140.3.6542

[25] Hernandez R, Martinez-Lara E, Del Moral ML, Blanco S, Canuelo A, et al (2004) Upregulation of endothelial nitric oxide synthase maintains nitric oxide production in the cerebellum of thioacetamide cirrhotic rats. Neuroscience 126: 879–887. 15207323 10.1016/j.neuroscience.2004.04.010

[26] Gabbott PL, Bacon SJ (1996) Localisation of NADPH diaphorase activity and NOS immunoreactivity in astroglia in normal adult rat brain. Brain Res 714: 135–144. 8861618 10.1016/0006-8993(95)01509-4

[27] Barna M, Komatsu T, Reiss CS (1996) Activation of type III nitric oxide synthase in astrocytes following a neurotropic viral infection. Virology 223: 331–343. 8806568 10.1006/viro.1996.0484

[28] Ben Achour S, Pascual O (2012) Astrocyte-neuron communication: functional consequences. Neurochem Res 37: 2464–2473. 22669630 10.1007/s11064-012-0807-0

[29] Theodosis DT, Poulain DA, Oliet SH (2008) Activity-dependent structural and functional plasticity of astrocyte-neuron interactions. Physiol Rev 88: 983–1008. 18626065 10.1152/physrev.00036.2007

[30] Halassa MM, Fellin T, Haydon PG (2007) The tripartite synapse: roles for gliotransmission in health and disease. Trends Mol Med 13: 54–63. 17207662 10.1016/j.molmed.2006.12.005

[31] Volterra A, Meldolesi J (2005) Astrocytes, from brain glue to communication elements: the revolution continues. Nat Rev Neurosci 6: 626–640. 16025096 10.1038/nrn1722

[32] Amin-Hanjani S, Stagliano NE, Yamada M, Huang PL, Liao JK, et al (2001) Mevastatin, an HMG-CoA reductase inhibitor, reduces stroke damage and upregulates endothelial nitric oxide synthase in mice. Stroke 32: 980–986. 11283400 10.1161/01.str.32.4.980

[33] Gertz K, Priller J, Kronenberg G, Fink KB, Winter B, et al (2006) Physical activity improves long-term stroke outcome via endothelial nitric oxide synthase-dependent augmentation of neovascularization and cerebral blood flow. Circ Res 99: 1132–1140. 17038638 10.1161/01.RES.0000250175.14861.77

[34] Endres M, Laufs U, Huang Z, Nakamura T, Huang P, et al (1998) Stroke protection by 3-hydroxy-3-methylglutaryl (HMG)-CoA reductase inhibitors mediated by endothelial nitric oxide synthase. Proc Natl Acad Sci U S A 95: 8880–8885. 9671773 10.1073/pnas.95.15.8880 PMC21171

[35] Cui X, Chopp M, Zacharek A, Zhang C, Roberts C, et al (2009) Role of endothelial nitric oxide synthetase in arteriogenesis after stroke in mice. Neuroscience 159: 744–750. 19154781 10.1016/j.neuroscience.2008.12.055 PMC2743134

[36] Calhoun ME, Jucker M, Martin LJ, Thinakaran G, Price DL, et al (1996) Comparative evaluation of synaptophysin-based methods for quantification of synapses. J Neurocytol 25: 821–828. 9023727 10.1007/BF02284844

[37] Alix JJ, Domingues AM (2011) White matter synapses: form, function, and dysfunction. Neurology 76: 397–404. 21263141 10.1212/WNL.0b013e3182088273

[38] Bakiri Y, Karadottir R, Cossell L, Attwell D (2011) Morphological and electrical properties of oligodendrocytes in the white matter of the corpus callosum and cerebellum. J Physiol 589: 559–573. 21098009 10.1113/jphysiol.2010.201376 PMC3055543

[39] Lo EH, Dalkara T, Moskowitz MA (2003) Mechanisms, challenges and opportunities in stroke. Nat Rev Neurosci 4: 399–415. 12728267 10.1038/nrn1106

[40] Dziewulska D, Rafalowska J, Podlecka A, Szumanska G (2004) Remote morphological changes in the white matter after ischaemic stroke. Folia Neuropathol 42: 75–80. 15266781

[41] Alix JJ (2006) Recent biochemical advances in white matter ischaemia. Eur Neurol 56: 74–77. 16946618 10.1159/000095543

[42] Pantoni L, Garcia JH, Gutierrez JA (1996) Cerebral white matter is highly vulnerable to ischemia. Stroke 27: : 1641–1646; discussion 1647. 10.1161/01.str.27.9.1641 8784142

[43] McIver SR, Muccigrosso M, Gonzales ER, Lee JM, Roberts MS, et al (2010) Oligodendrocyte degeneration and recovery after focal cerebral ischemia. Neuroscience 169: 1364–1375. 20621643 10.1016/j.neuroscience.2010.04.070 PMC3789594

[44] Shi X, Kang Y, Hu Q, Chen C, Yang L, et al (2010) A long-term observation of olfactory ensheathing cells transplantation to repair white matter and functional recovery in a focal ischemia model in rat. Brain Res 1317: 257–267. 20043890 10.1016/j.brainres.2009.12.061

[45] Johnston MV (2009) Plasticity in the developing brain: implications for rehabilitation. Dev Disabil Res Rev 15: 94–101. 19489084 10.1002/ddrr.64

[46] Pozzo-Miller LD, Gottschalk W, Zhang L, McDermott K, Du J, et al (1999) Impairments in high-frequency transmission, synaptic vesicle docking, and synaptic protein distribution in the hippocampus of BDNF knockout mice. J Neurosci 19: 4972–4983. 10366630 10.1523/JNEUROSCI.19-12-04972.1999 PMC6782660

[47] Madinier A, Bertrand N, Rodier M, Quirie A, Mossiat C, et al (2013) Ipsilateral versus contralateral spontaneous post-stroke neuroplastic changes: involvement of BDNF? Neuroscience 231: 169–181. 23219910 10.1016/j.neuroscience.2012.11.054

[48] Liu HS, Shen H, Harvey BK, Castillo P, Lu H, et al (2011) Post-treatment with amphetamine enhances reinnervation of the ipsilateral side cortex in stroke rats. Neuroimage 56: 280–289. 21349337 10.1016/j.neuroimage.2011.02.049 PMC3070415

[49] Griesbach GS, Hovda DA, Molteni R, Wu A, Gomez-Pinilla F (2004) Voluntary exercise following traumatic brain injury: brain-derived neurotrophic factor upregulation and recovery of function. Neuroscience 125: 129–139. 15051152 10.1016/j.neuroscience.2004.01.030

[50] Medina DL, Sciarretta C, Calella AM, Von Bohlen Und Halbach O, Unsicker K, et al (2004) TrkB regulates neocortex formation through the Shc/PLCgamma-mediated control of neuronal migration. EMBO J 23: 3803–3814. 15372074 10.1038/sj.emboj.7600399 PMC522798

[51] Bergami M, Santi S, Formaggio E, Cagnoli C, Verderio C, et al (2008) Uptake and recycling of pro-BDNF for transmitter-induced secretion by cortical astrocytes. J Cell Biol 183: 213–221. 18852301 10.1083/jcb.200806137 PMC2568011

[52] Kovalchuk Y, Holthoff K, Konnerth A (2004) Neurotrophin action on a rapid timescale. Curr Opin Neurobiol 14: 558–563. 15464888 10.1016/j.conb.2004.08.014

[53] Juric DM, Miklic S, Carman-Krzan M (2006) Monoaminergic neuronal activity up-regulates BDNF synthesis in cultured neonatal rat astrocytes. Brain Res 1108: 54–62. 16828062 10.1016/j.brainres.2006.06.008

[54] Rudge JS, Pasnikowski EM, Holst P, Lindsay RM (1995) Changes in neurotrophic factor expression and receptor activation following exposure of hippocampal neuron/astrocyte cocultures to kainic acid. J Neurosci 15: 6856–6867. 7472443 10.1523/JNEUROSCI.15-10-06856.1995 PMC6577973

[55] Kim H, Li Q, Hempstead BL, Madri JA (2004) Paracrine and autocrine functions of brain-derived neurotrophic factor (BDNF) and nerve growth factor (NGF) in brain-derived endothelial cells. J Biol Chem 279: 33538–33546. 15169782 10.1074/jbc.M404115200

[56] Wang H, Ward N, Boswell M, Katz DM (2006) Secretion of brain-derived neurotrophic factor from brain microvascular endothelial cells. Eur J Neurosci 23: 1665–1670. 16553631 10.1111/j.1460-9568.2006.04682.x

[57] Huang EJ, Reichardt LF (2003) Trk receptors: roles in neuronal signal transduction. Annu Rev Biochem 72: 609–642. 12676795 10.1146/annurev.biochem.72.121801.161629

[58] Du Y, Fischer TZ, Lee LN, Lercher LD, Dreyfus CF (2003) Regionally specific effects of BDNF on oligodendrocytes. Dev Neurosci 25: 116–126. 12966210 10.1159/000072261

[59] Du Y, Lercher LD, Zhou R, Dreyfus CF (2006) Mitogen-activated protein kinase pathway mediates effects of brain-derived neurotrophic factor on differentiation of basal forebrain oligodendrocytes. J Neurosci Res 84: 1692–1702. 17044032 10.1002/jnr.21080

[60] Dugas JC, Tai YC, Speed TP, Ngai J, Barres BA (2006) Functional genomic analysis of oligodendrocyte differentiation. J Neurosci 26: 10967–10983. 17065439 10.1523/JNEUROSCI.2572-06.2006 PMC6674672

